# Effects of polymorphisms in ovine and caprine prion protein alleles on cell-free conversion

**DOI:** 10.1186/1297-9716-42-30

**Published:** 2011-02-15

**Authors:** Martin Eiden, Elizabeth Ortega Soto, Thomas C Mettenleiter, Martin H Groschup

**Affiliations:** 1 Institute for Novel and Emerging Infectious Diseases at the Friedrich-Loeffler-Institut, Federal Research Institute for Animal Health, D-17493 Greifswald-Insel Riems, Germany; 2 Institute of Molecular Biology at the Friedrich-Loeffler-Institut, Federal Research Institute for Animal Health, D-17493 Greifswald-Insel Riems, Germany

## Abstract

In sheep polymorphisms of the prion gene (*PRNP*) at the codons 136, 154 and 171 strongly influence the susceptibility to scrapie and bovine spongiform encephalopathy (BSE) infections. In goats a number of other gene polymorphisms were found which are suspected to trigger similar effects. However, no strong correlation between polymorphisms and TSE susceptibility in goats has yet been obtained from epidemiological studies and only a low number of experimental challenge data are available at present. We have therefore studied the potential impact of these polymorphisms in vitro by cell-free conversion assays using mouse scrapie strain Me7. Mouse scrapie brain derived PrP^Sc ^served as seeds and eleven recombinant single mutation variants of sheep and goat PrP^C ^as conversion targets. With this approach it was possible to assign reduced conversion efficiencies to specific polymorphisms, which are associated to low frequency in scrapie-affected goats or found only in healthy animals. Moreover, we could demonstrate a dominant-negative inhibition of prion polymorphisms associated with high susceptibility by alleles linked to low susceptibility in vitro.

## Introduction

Prion diseases include scrapie in sheep, bovine spongiform encephalopathy (BSE) in cattle and Creutzfeldt-Jacob-Disease (CJD) in humans and are characterized by the conversion of cellular prion protein (PrP^C^) into an abnormal pathological isoform called PrP^Sc^. In the course of the conversion the prion protein is transformed from a predominantly α-helical structure into a β-sheet rich conformation. Disease development is characterized by an accumulation of PrP^Sc ^followed by neuronal degeneration and CNS dysfunction. The PrP gene (*PRNP*) polymorphisms have been associated with distinct disease phenotypes in humans and sheep. These phenotypes include differences in the incubation period, PrP^Sc ^deposition pattern, pathogenesis and even clinical signs following the infection with a given strain or isolate. Sheep carrying the *PRNP *polymorphisms valine (V) or alanine (A) at codon 136 (136V and 136A) are highly susceptible to classical scrapie, while the exchange of arginine (R) to histidine at codon 154 (154H) is linked to low scrapie susceptibility [1,2]. Interestingly, this allele is associated with high susceptibility to atypical/Nor98 scrapie in sheep and goats [3,4] and short incubation periods in BSE infected sheep [5]. The exchange of glutamine (Q) to arginine (R) at codon 171 (171R) induces a nearly resistant phenotype [6,7]. Based on these findings a sheep breeding program in Europe was carried out to propagate the 171R allele [8].

Genetic analysis of the goat PRNP revealed 42 polymorphisms in the open reading frame including silent mutations [9]. Some of these polymorphisms are associated with changes in the susceptibility to scrapie: At codon 142 an exchange from isoleucine (I) to methionine (M) (142M) prolongs the incubation time after a challenge with scrapie and BSE prions [10]. A reduced susceptibility to natural scrapie has also been reported for goats carrying arginine (R) at codon 143 (143R) and histidine (H) at codon 154 (154H) [11] in the PRNP gene as well as for goats with glutamine (Q) at codon 211 (211Q) [12]. Recently, two novel polymorphism were found at position 146, harboring serine (S) or aspartic acid (D) (146S, 146D), which were linked to resistance against scrapie [13]. In addition, lysine (K) at codon 222 (222K) is only found in healthy goats and is associated with low susceptibility to scrapie [14]. The caprine wildtype allele contained isoleucin (I) at position 142, histidine (H) at position 143, asparagine (N) at position 146, arginine (R) at positions 151 and 211 and glutamine (Q) at position 222 and was subsequently denoted IHNRRQ. Because of the variety of mutations in the caprine *PRNP *gene, their impact on the susceptibility is difficult to ascertain experimentally in vivo.

To analyze the effect of single amino acid substitutions on the convertibility of ovine and caprine prion protein variants, an in vitro approach was therefore used in this study. Several in-vitro assays were reported before. The first assay that was reported used purified and radiolabeled PrP^C ^molecules, which were incubated with PrP^Sc ^seeds and converted into proteinase K resistant PrP^res ^fragments [15-17]. This type of assay was intensively used to analyze interspecies and intraspecies transmission barriers [18]. Recently, a modified cell-free conversion assay was established which uses both prion components under equimolar and semi-native conditions [19]. Another assay was published which dealt with aggregation and fibrillation of recombinant prion protein in the absence of PrP^Sc ^seeds [20]. However, the infectious nature of the so-called "synthetic prions" still remains enigmatic [21].

In this study we used a cell-free conversion assay to evaluate *PRNP *polymorphisms in sheep and goats in vitro. We therefore generated 11 bacterial prion variants haboring different ovine and caprine PrP^C ^polymorphisms. The conversion was carried out with mouse passaged scrapie strain Me7, which originated from classical scrapie isolates [22]. Our results provide important clues regarding impacts of aminoacid exchange on the conversion of prion protein and demonstrate a high accordance between our findings in vitro and epidemiological data.

## Material and methods

### Mutation of *PRNP*

Ovine prion alleles were amplified from the ORF 23-234 by PCR using primers ov1 (5'-CCGGATCCAAGAAGCGACC AAAACC-3') and ov2 (5'-CCAAGCTTCTAGCTGGATC TTCTCCCG-3'). As template we used genomic DNA from genotyped homozygous sheep alleles (136A, 154H and 171R) [23]. Caprine polymorphisms were generated by the substitu-tion of amino acids at residues 142, 143, 146, 151, 211 and 222 using the ovine 136A allele as a template. The caprine mutations were carried out by a two step PCR which first uses internal primers for the particular substitution and then external primers ov1/ov2 for the amplification of the mutant template. The 142M mutation was introduced by the use of a forward primer (forv) 5'-AGG CCT CTT ATG CAT TTT GGC-3' and a reverse primer (rev) 5'-GCC AAA ATG CAT AAG AGG CCT-3', the 143R mutation with forv 5'-CCT CTT ATA CGT TTT GGC AAT G-3' and rev 5'-C AAT GCC AAA ACG TAT AAG AGG-3' primer, the 146S with forv 5'-CAT TTT GGC TCT GAC TAT GAG-3' and rev 5'-CTC ATA GTC AGA GCC AAA ATG-3' primer, the 146D with forv 5'-CAT TTT GGC GAT GAC TAT GAG-3' and rev 5'-CTC ATA GTC ATC GCC AAA ATG-3' primer, the 151H mutation with forv 5'-C TAT GAG GAC CAT TAC TAT CG-3' and rev 5'-CG ATA GTA ATG GTC CTC ATA G-3' primer, 211Q with forv 5'-GATA ATG GAG CAA GTG GTG GAG-3' and rev 5'-CTC CAC CAC TTG CTC CAT TAT C-3' primer and the 222K mutation with rev 5'-GG AAC CTT CTA ACT TGC CCC CCT TTG GTA ATA AGC CTG GGA TTC TCT CTT GTA CTG G-3' primer.

### Expression and purification of recombinant PrP

The amplified gene was cloned via *Bam*HI and *Hind*III restriction sites into the vector pQE-40 (Qiagen, Hilden, Germany) which harbors an N-terminal histidin (his-)-tag. Expression and purification was described previously [19]. In brief, purification was carried out under denaturating conditions over an N-terminal His-tag using a Ni-NTA-column according to standard procedures. Refolding was accomplished by dialysis in the corresponding refolding buffer [24]. CD spectra of all constructs displayed a high content of α-helical structure, which indicates proper folding of the proteins (data not shown).

### Cell-free conversion assay

Cell-free conversion was carried out by mixing 400 ng of recombinant prion protein with 400 ng purified PrP^Sc ^seeds (mouse passaged scrapie strain Me7) in a modified conversion buffer (50 mM citrate pH 6.0, 200 mM KCl, 5 mM MgCl_2_, 1.25% N-lauroylsarcosine and 2.0 μg/μL suramine). After three days of incubation at 37 °C the samples were treated with Proteinase K (PK) for 1 h at 37 °C at a final concentration of 30 μ/mL (in 50 mM Tris/HCl, pH 7.4, 150 mM NaCl). The reaction was stopped with 10 mM Phenylmethylsulfonylfluorid (PMSF). Thyreoglobuline was added to the samples, which were subsequently incubated in methanol at -20 °C for at least 1 h [19]. Proteins were pelleted by centrifugation at 13 000 rounds per minute (rpm) for 15 min and subjected to sodium dodecyl sulphate-polyacrylamide gel electrophoresis (SDS-PAGE). PrP^Sc ^was purified from terminally scrapie diseased mouse brains (mouse scrapie Me7) according to a previous publication [19]. Detection of the newly converted PrP^res ^fragments was accomplished by immunoblotting, using mab P4 [25] as detection antibody and a chemiluminiscence visualisation. Chemiluminescence signals were detected using the BioRad VersaDoc™ photo imaging system and analysed with the Quantity One quantification software (BioRad, Munich, Germany). All constructs were analysed in a cell-free conversion assay and the conversion rates of PrP variants were calculated on the basis of the generated PrP^res ^fragments. Relative conversion rates of ovine PrP variants were calculated in relation to the ovine 136A reference allele (*n *= 4) and the goat derived variants in relation to the caprine IHNRRQ reference allele (*n *= 8). Statistical analysis was performed with unpaired student *t*-test and statistical significance was expressed as follows: ns: not significant, *: *p *< 0.05, **: *p *< 0.01, ***: *p *< 0.001. In the case of unequal statistical spread of variances, Student's *t*-test was supplemented by the Welch correction (Welch's *t*-test).

## Results

**Figure 1:**
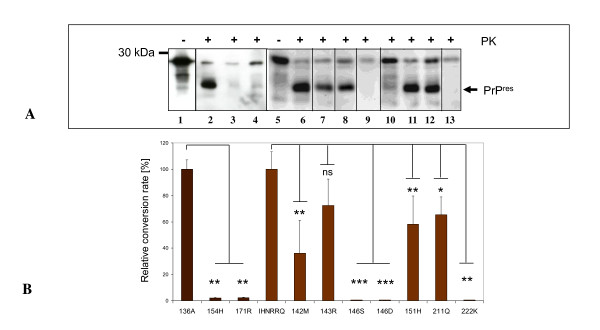
**Cell-free conversion of ovine and caprine PrP^C ^variants by scrapie prions**. **(A) **Detection of cell-free converted PrP^res^. Non-proteinase K (PK) treated wildtype 136A and wildtype IHNRRQ samples are shown in lane 1 and lane 5 respectively. PrP^res ^fragments of 136A (lane 2) and its variants 154H (lane 3) and 171R (lane 4) are depicted. PrP^res ^fragments of caprine IHNRRQ (lane 6) and its variants 142M (lane 7), 143R (lane 8), 146S (lane 9), 146D (lane 10), 151H (lane 11), 211R (lane 12) and 222K (lane 13). The ovine and caprine PrP was detected using monoclonal antibody (mab) P4. Molecular mass marker is indicated on the left. Arrow indicates PrP^res ^fragments. **(B) **Mean relative conversion efficiencies (± standard error of the mean, SEM) for each set of conversion reaction. Relative conversion rates of ovine PrP variants were calculated in relation to the ovine 136A reference allele and the goat derived variants in relation to the caprine IHNRRQ reference allele. Bars depict the SEM of at least 4 reactions. The differences were analyzed by unpaired student *t*-test. *: *p *< 0.05; **: *p *< 0.01; ***: *p *< 0.001. ns: not significant.

Figure 1 shows the conversion of recombinant prokaryotic ovine prion protein (136A allele) into its PK resistant form, PrP^res ^(Figure 1A, lane 2). The shift of approximately 6-7 kDa (in comparison to the nontreated control) (Figure 1A, lane 1) results from the partial resistance of PrP^res^, i.e. removal of the nonstructured N-terminus. The relative ratio of converted PrP^C ^was approx. 5% of the PrP^C ^that was initially used in the reaction. Conversions were carried out with mouse passaged scrapie strain Me7 as described before [19]. The physiological relevance of the assay was demonstrated with ovine PrP polymorphisms 154H and 171R, which strongly affect the scrapie susceptibility in sheep. In vitro, the conversion of the 154H (Figure 1A, lane 3) and 171R alleles were almost completely absent (Figure 1A, lane 4) compared to 136A conveying susceptibility in vivo (Figure 1A, lane 2). The relative conversion rates were 1.9% and 2.14% respectively as shown in Figure 1B.

**Figure 2:**
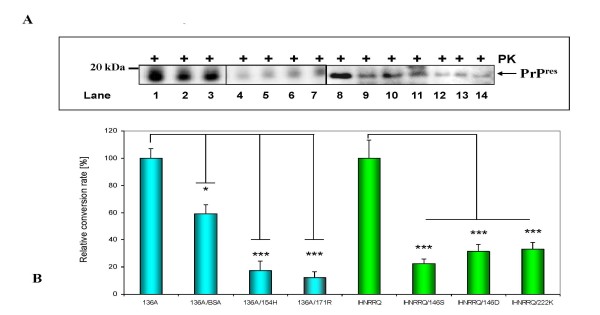
**Dominant inhibition of the cell-free conversion by alleles associated with low susceptibility**. **(A) **PrP^res ^fragments generated from 136A (lane 1), a 1:1 mxiture of 136A with BSA (lanes 2-3), a 1:1 mixture of 136A with 171R (lanes 4-5), a 1:1 mixture of 136A with 154H (lanes 6-7). PrP^res ^fragments derived from IHNRRQ (lane 8), a 1:1 mixture of IHNRRQ with 146S (lanes 9-10), a 1:1 mixture of IHNRRQ with 146D (lanes 11-12) and a 1:1 mixture of IHNRRQ with 222K (lanes 13-14). Detection was carried out using mab P4. Arrow indicates PrP^res ^fragments. **(B) **Mean relative conversion efficiencies (± SEM) for each set of at least four conversion reactions. Relative conversion rates were calculated in relation to the ovine 136A reference allele and the caprine IHNRRQ reference allele. The differences were analyzed by unpaired student *t*-test. *: *p *< 0.05; **: *p *< 0.01; ***: *p *< 0.001. ns: not significant.

To examine, whether the alleles 154H or 171R, which are linked to low susceptibility, had an inhibitory effect on the cell-free conversion of the ovine 136A allele, 1:1 mixtures of 136A with variants 154H or 171R (corresponding to heterozygous 136A/154H and 136A/171R animals) were carried out. Both combinations 136A/171R (Figure 2A, lanes 4-5) and 136A/154H (Figure 2A, lanes 6-7) showed a clear reduction to 17.5% (154H) and 12.3% (171R) in the conversion rates (Figure 2B) compared to 136A alone (Figure 2A, lane 1, Figure 2B). As a control a 1:1 mixture of 136A with bovine serum albumine as additive was used (retaining the same total amount of protein of the sample) in order to analyse the conversion rate of 50% of the original 136A PrP. The determined reduction was 59%, which correlates to the half of the amount of 136A (Figure 2A, lanes 2-3, Figure 2B). The significant reduction by 154H and 171R allele, which exceeded 50% by far, underlines their direct interference with the 136A allele and definitely shows an inhibitory effect of 171R and 154H alleles over the 136A alleles at the protein level.

The same studies were carried out with the caprine PrP variants. The conversion of the wildtype allele IHNRRQ with the mouse-passaged scrapie strain Me7 was confirmed by the formation of a PK-resistant PrP^res ^fragment (Figure 1A, lane 6), which harboured a shift in the molecular mass of about 6-7 kDa in contrast to the untreated control (Figure 1A, lane 5). Amino acid exchanges 142M (Figure 1A, lane 7), R151H (Figure 1A, lane 11) and 211Q (Figure 1A, lane 12) resulted in significantly reduced conversion rates of up to 34.9% (for 142M), 56.9% (for R151H) and 65.4% (for 211Q) in comparison to the IHNRRQ allele alone (Figure 1A, lane 6). Only the 143 allele showed a non significant reduction to 71.1% (Figure 1A, lane 8). Exchanges at position 146 (146D, 146S) and 222 (222K) inhibited a conversion completely (Figure 1A, lanes 9, 10, 13). The relative conversion rates are displayed in Figure 1B. An incomplete proteolysis of full-length PrP by Proteinase K was observed infrequently (e.g. Figure 1A lane 10), and is a concession to using native buffer conditions and the absence of denaturants like urea or guanidinium hydrochloride.

Possible dominant-negative inhibitions were also analyzed in 1:1 mixtures of the caprine IHNRRQ allele co-incubated with the alleles 146S (IHNRRQ/146S), 146D (IHNRRQ/146D) and 222K (IHNRRQ/222K), which are associated with resistance to scrapie. The IHNRRQ conversion was significantly inhibited by 146S (Figure 2A, lanes 9-10), 146D (Figure 2A, lanes 11-12) and 222K (Figure 2A, lanes 13-14) with corresponding relative conversion rates of 22.4%, 31.4% and 33.1% respectively (Figure 2B). Again, the significant reduction of more than 50% indicates the direct interference of conversion incompetent alleles with the wild-type IHNRRQ allele, leading to dominant-negative inhibition.

## Discussion

Cell-free conversion assays were widely used for the determination of PrP conversion efficiencies and evaluation of species barriers in vitro. Here we report the conversion of ovine and caprine polymorphisms in vitro and their association with susceptibility to scrapie in vivo. The used cell-free conversion assay is based on the conversion of recombinant ovine and caprine PrP by a PrP^Sc ^seed and involves antibodies, that discriminate between newly converted PrP^res ^and the used PrP^Sc ^seed via a species specific epitope. We therefore required a scrapie strain which was passaged in another species. We selected the mouse passaged scrapie strain Me7 for our studies, since it was isolated from various classical scrapie cases. Moreover, Me7 retained its biological and physicochemical characteristics during multiple passages in a variety of different species [22,26-28]. Although the conversion of ovine PrP by mouse passaged strain Me7 crosses the species barrier, the newly converted recombinant ovine PrP^res ^displays the same biochemical characteristics as PrP^Sc ^derived from original scrapie. This includes partial PK-resistance, detection by mab P4 as well as mab L42 and a shift in the molecular mass of the unglycosylated fragment compared to BSE derived fragments (data not shown). The available data suggest that the biological properties of the newly formed PrP^res ^molecules are not modified after conversion with mouse adapted strains and that this model represents a suitable system to study ovine and caprine PrP polymorphisms on scrapie susceptibility in vitro.

Molecular structure analysis studies were obtained primarily for ovine PrP^C ^variants and compared to corresponding convertibility data. The absence of conversion of the 171R allele was explained by the exchange of glutamine (Q) by the basic residue arginine (R) at position 171, which interferes with the formation of the extended β-sheet rich structure during the conversion [29]. In addition, using spectroscopic analysis, it could be demonstrated that the 171R conformation was less stable than the 136A conformation at physiological temperatures. The low conformational stability inhibited amyloid fibril formation [30]. In the case of the 154H allele, the exchange of arginine to histidine at position 154 was perturbing the α-helix 1 by disrupting the salt-bridge between residues D150 and R154 (positions according to human PrP) [31]. Although CD-spectra of different alleles showed similar α-helical structures the exchange of one amino acid in the 171R and 154H allele caused a reduced thermodynamic and thermal stability in the protein [32].

This is similar to the situation in vivo: expression of the 171R allele is related to scrapie resistance, and the 154H allele causes a reduced susceptibility to the disease. In contrast the 136A genotype was found to be highly susceptible, when exposed to natural or experimental classical scrapie [33]. The homozygous genotype 154H/154H was proposed to be nearly as protective against scrapie as 171R/171R [23]. 154H was also associated with resistance to natural scrapie in Cypriot goats [13]. However, the influence of the 154H allele on the scrapie susceptibility is ambiguous, which is highlighted by the occurrence of scrapie positive sheep with a homozygous 154H/154H genotype in a Merinoland sheep flock [34]. Even in sheep, carrying the 171R/171R genotype, which is generally regarded as highly resistant to classical scrapie, two classical scrapie cases were reported [35]. Both alleles, however, are linked to susceptibility for atypical/Nor98 scrapie in affected heterozygous animals [36,37]. Moreover, 171R/171R genotypes [38] and 154H/154H genotypes [39] are susceptible to BSE after experimental infection. These data indicate that the influence of both alleles to scrapie susceptibility displays a complex interplay with different TSE strains and probably depends on additional genetic and epidemiological risk factors.

Co-incubation of the 171R and 154H variant with the 136A allele, termed 136A/171R and 136A/154H, resulted in a significantly reduced conversion rate in contrast to the single 136A genotype. A similar effect has been observed with hamster allele 172R, which inhibits the conversion of wild type hamster PrP in vitro [40]. According to the stone fence model [41], the loss of conversion efficiency can be explained by the incorporation of conversion incompetent 171R or 154H molecules into the 136A aggregates, which delay or block fibril elongation. The interference of alleles linked to high susceptibility with alleles linked to low susceptibility in vitro is in good accordance with the situation in vivo. Sheep carrying the 136A/154H or the 136A/171R allele show a reduced susceptibility to scrapie compared to the 136A/136A allele [2]. The dominant-negative inhibition works therefore on protein level and is probably not caused by preferential expression of the susceptible alleles. This could be demonstrated by assessment of PrP transcripts from heterozygous 136A/171R sheep and the confirmation of equal use of both alleles [42]. These results also argument also against the existence of the proposed factor X [43]. However, the age related increase of 136A/154H scrapie cases [2] highlights the erratic role of 154H polymorphism and suggests the involvement of additional factors.

Due to a limited number of goats, which harbour disease associated polymorphisms, a significant correlation of specific polymorphisms to susceptibility is difficult to obtain in vivo. The 142M polymorphism is found with a low frequency in the goat population, but experimental infection of 142M goats with scrapie and BSE strains showed an increased incubation period [10]. This is in agreement with the reduced convertibility of the recombinant 142M allele in vitro. A similar correlation was found at codon 143. Studies on the relative scrapie incidence in goats carrying different PrP genotpes in Greek goat herds revealed a moderately protective effect of the 143R polymorphism [11]. We have observed a similar effect in the here reported in vitro studies. A similar protective effect may also exist for the R151H polymorphism in Cypriot goats [13,44]. Another polymorphism (211Q) in French goats is also suspected to reduce the scrapie susceptibility [12]. This is consistent with our findings, in which the exchange of arginine (R) to glutamine (Q) at position 211 in recombinant PrP caused a reduced conversion rate.

Two novel polymorphisms at position 146 (146D and 146S) were found in scrapie-negative animals in Cypriot goat herds [44]. The protective effects of exchanges from asparagine (N) to aspartic acid (D) or serine (S) were confirmed in the cell-free conversion by the complete absence of PrP^res ^fragments of the respective PrP^C ^variant. The inhibitory potential of these variants was confirmed by dominant-negative effects in vitro, where co-incubation with the susceptible IHNRRQ allele caused a significant decrease in the conversion rate for both the IHNRRQ/146D and the IHNRRQ/146S variants.

An exchange from glutamine (Q) to lysine (K) at position 222 (222K) revealed another protective polymorphism in goats. Looking at scrapie outbreaks in Italian goats, this polymorphism was found only in healthy animals [14]. The conversion of recombinant 222K allele was completely abolished in vitro and significantly reduced the conversion rate of the susceptible IHNRRQ allele during co-incubation. Similar effects were found in human PrP, where the E219K allele was reported to protect against Creutzfeldt-Jakob-Disease [45]. In addition, the influence of the Q218K variant of the mouse prion protein was analysed in vitro and found to significantly reduce aggregation kinetic and induce a dominant-negative effect on murine wildtype prion protein [46]. This way, insertion of an additional positive charge at position 222 of the caprine prion protein is assumed to interfere with PrP^C^/PrP^Sc ^interaction.

In summary, the here reported in vitro conversion studies support the association of specific ovine and caprine *PRNP *polymorphisms with a reduced classical scrapie susceptibilities in vivo. Moreover we could demonstrate an interference of susceptible PrP alleles by co-incubation with inhibitory alleles in vitro, which apparently corresponds to prolonged incubation times of heterozygous animals in vivo.

## Authors' contributions

ME and EOS carried out the experimental work and performed the statistical analysis. TCM reviewed the data interpretation. MHG and ME designed this study, interpreted the results and wrote the manuscript. All authors read and approved the final manuscript.

## Competing interests

The authors declare that they have no competing interests.
